# Development of a Core Outcome Set for Intervention Studies in Adults With Laryngotracheal Stenosis

**DOI:** 10.1002/lary.32262

**Published:** 2025-05-12

**Authors:** Gemma M. Clunie, Amy Freeman‐Sanderson, Chadwan Al‐Yaghchi, Justin W. G. Roe, Caroline Alexander, Guri Sandhu, Alison McGregor, Louise Rose

**Affiliations:** ^1^ Imperial College London London UK; ^2^ Imperial College Healthcare NHS Trust London UK; ^3^ Graduate School of Health, University of Technology Sydney Chippendale New South Wales Australia; ^4^ Royal Prince Alfred Hospital Sydney New South Wales Australia; ^5^ Critical Care Division The George Institute for Global Health, Faculty of Medicine, UNSW Sydney Sydney Australia; ^6^ Australian and New Zealand Intensive Care Research Centre (ANZIC‐RC), school of Public Health and Preventive Medicine Monash University Melbourne Australia; ^7^ Faculty of Nursing, Midwifery and Palliative Care, King's College London London UK

**Keywords:** clinical trials, core outcome set, laryngotracheal stenosis

## Abstract

**Objective:**

Adult acquired laryngotracheal stenosis (LTS) is a chronic condition with heterogeneous treatment options and a significant symptom burden. Synthesis of data across research studies to guide clinical decision‐making is challenging due to inconsistent outcome selection and use of unvalidated measures. Our objective was to establish a core outcome set (COS) for studies of LTS interventions in adults.

**Methods:**

We conducted a two‐round modified e‐Delphi study. We reviewed published systematic reviews and qualitative studies to inform the inclusion of 42 outcomes in the first e‐Delphi round, with 10 additional outcomes added by participants for the second voting round. The international expert panel included clinicians, researchers, and people living with LTS. We held two consensus meetings and a final voting round.

**Results:**

The first e‐Delphi round involved 1067 participants from multiple stakeholder groups, with 575 participants voting in the second. Seventeen participants participated in the consensus meetings. The final COS included seven outcomes: (1) Level of breathlessness, (2) Ability to generate audible voice, (3) Ability to manage/clear mucus, (4) Ability to eat and drink, (5) Health‐related quality of life, (6) Emotional and mental health symptoms, and (7) Frequency of treatment.

**Conclusion:**

By using a rigorous Delphi process informed by multiple stakeholder groups, we gained consensus on seven core outcomes for inclusion in future research relating to LTS. Use of this COS will standardize outcomes measured in future research studies, ensuring they are comparable. Future work is required to identify the best way to measure these outcomes to fully operationalize this COS.

**Level of Evidence:**

Level V.

## Graphical abstract

We used a rigorous two round Delphi process informed by multiple stakeholder groups to gain consensus on seven core outcomes for inclusion in future research relating to adults with laryngotracheal stenosis. Use of this core outcome set will standardise outcomes measured in future research studies, ensuring they are comparable.

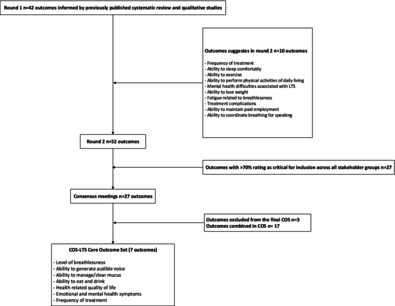

## 1 Introduction

Adult acquired laryngotracheal stenosis (LTS) is a rare chronic condition defined by airway narrowing from the supraglottis to the carina [1]. Causes are heterogeneous and include iatrogenic injury, for example, intubation, trauma, autoimmune conditions, and idiopathic subglottic stenosis (iSGS), a fibroinflammatory process predominantly affecting white women aged between 40 and 60 [2, 3, 4]. Treatment options range from repeated endoscopic procedures to improve airway lumen diameter to complex reconstructive procedures. LTS and its treatments have a significant symptom burden impacting breathing, voice, and swallowing, with some patients requiring long‐term tracheostomy. These functional difficulties lead to limitations on activities and participation, impacting quality of life [5]. Due to the heterogeneity of the condition, it is challenging to identify the health economic cost of LTS; however, mean annual healthcare costs are estimated at USD$4,081 per patient, with reconstructive procedures costing USD$8,584. Costs are cumulative over time, with intubation‐related disease accruing more annual costs than the idiopathic subtype [6] although a recent study demonstrated that earlier intervention can reduce this burden [7]. Symptom cost, impact on personal activities, and health‐related quality of life for people with LTS are currently unknown.

Research to date has primarily focused on breathing outcomes following specific surgical approaches. However, there is increasing acknowledgment of the need to understand functional outcomes [5, 8]. The use of a core outcome set (COS) in intervention studies reduces heterogeneity of outcome reporting and allows for improved standardization across research studies. This supports evidence‐based practice for clinicians and allows study findings to be combined to maximize impact. However, there is no consistent approach to outcome selection within LTS studies, and the most commonly used outcome measures are not validated for this population. This makes the synthesis of findings between LTS studies challenging and limits comparison and detailed analysis of treatment benefit [9, 10]. This creates a significant barrier to developing evidence‐based guidelines for clinicians working with LTS patients.

The goal of COS development is to standardize outcome reporting across studies by identifying outcomes perceived as fundamental for measurement in all trials of a specific interest area [11]. The Core Outcome Measures in Effectiveness Trials (COMET) initiative has endorsed Delphi consensus methods as part of the protocol for the development of a COS and provides examples of this process [11]. In this study, our aim was to establish a COS for studies of interventions designed to assess and rehabilitate voice, swallowing, mucus, and cough in adults with LTS, alongside the more commonly reported breathing outcomes.

## 2 Materials and Methods

We used the COMET guidelines [11] to guide this COS development study. We report our methods and findings using the Core Outcome Set‐STAndards (COS‐STAD—see Data S1) [12]. The COS was registered on the COMET Database in May 2023 (registration number 2677). Ethical approval (minimal risk registration number: MRA‐22/23–36,472) was granted by the Kings College London research ethics committee. To provide a systematic assessment of the study, we have completed a quality appraisal [13] (see Data S2).

### 2.1 E‐Delphi Methods

Item generation for the COS was informed by a previously published systematic review [14] and qualitative studies that included consideration of outcomes for LTS [15, 16, 17]. All items were initially extracted by one author (GC), with two further authors (AFS, LR) reviewing the outcome list and their definitions. Discrepancies or disagreements were settled via discussion. Items on the final list were then mapped according to the COMET taxonomy [18]. For example, the outcome “Ability to use voice at work” was mapped to the domain “Life impact.” This process was completed by one author (GC) and reviewed by two further authors (CA, LR), with disputes resolved by discussion. This list was then reviewed by a patient advisor to ensure the outcomes corresponded to their experience of living with LTS. The final list of outcomes, including lay descriptions to clarify the definition of each outcome, formed the e‐Delphi round 1, which was piloted with key stakeholders, including clinicians, researchers, and people living with LTS, to receive feedback on wording, comprehensibility, and readability. Feedback was then incorporated to produce the final version of the e‐Delphi round 1 questionnaire. We administered the e‐Delphi rounds using custom Delphi Manager software, version 5.0 (COMET Initiative, Liverpool, UK).

### 2.2 Participants and Recruitment

We used the following recruitment methods: snowball sampling via the personal and professional networks of the research team, including British Laryngological Association membership (BLA), European Laryngological Society (ELS), North American Airway Collaborative (NoAAC), and Royal College of Speech and Language Therapists (RCSLT), and via the email distribution list and online iSGS support group managed by the patient advisor to the project. Flyers inviting potential participants to take part were sent by email. We aimed to recruit four key stakeholder groups as follows: (1) *Clinicians (no research)*, comprising surgeons, nurses, allied health professionals, and others who manage patients with LTS as part of their clinical practice; (2) *Researchers not engaged in clinical practice*, comprising professionals conducting research into LTS; (3) C*linician researchers*, comprising individuals conducting research who also work clinically with people with LTS; and (4) *People living with LTS or a family member/friend* comprising patients and caregivers.

Inclusion criteria were as follows: ability to follow written and spoken English and willingness to participate. In addition, clinicians or researchers were defined as registered with their regulatory body, with at least 2 years of clinical experience caring for people with LTS and/or with professional standing/published research in LTS. For people living with LTS, we included those who self‐reported their diagnosis. To be able to participate in the Delphi consensus meetings, participants had to have voted in both e‐Delphi rounds.

### 2.3 Delphi Rounds

Participants received an online invitation via the Delphi Manager software to complete the first Delphi survey. Participants were asked to select their stakeholder group and provide demographic information: country of residence, age category, self‐reported year diagnosed with LTS (if a patient), profession, and years of experience working with LTS (if a clinician/researcher). Participants were then asked to score the importance of each of the 42 outcomes for COS inclusion using the COMET's recommended Grading of Recommendations Assessment, Development, and Evaluations (GRADE) Scale [19]. This is a 9‐point Likert scale, with 1–3 indicating not important, 4–6 important but not critical, and 7–9 critical for inclusion. Participants were also able to select *unable to score* and offer additional outcome suggestions. The outcome domains were randomized by the software to avoid presentation bias.

Following round 1, we calculated the median and interquartile range (IQR) of GRADE importance scores and the proportion of participants rating each outcome with scores of 1–3 (not important), 4–6 (important but not critical), and 7–9 (critically important) separately for each stakeholder group and across all stakeholder groups. Additional outcomes suggested by participants were reviewed, duplicates removed, and outcomes suitably reworded prior to their inclusion in round 2.

For round 2 participants were provided written and visual representations of their previous round 1 scores, as well as the summarized scores of the participants in each group described above (histogram plots). They were then asked to re‐score outcome importance and provide scores for the additional outcomes. If participants altered their scoring so that it moved to a new GRADE category, for example, “not important” to “important but not critical,” they were asked to provide a reason for the change. All outcomes included in the Delphi were voted on twice using the methods described. For each round, the Delphi Manager software was used to send three email reminders every 2 weeks for 6 weeks.

### 2.4 Delphi Consensus Meetings

All participants who completed all rounds of voting were invited by email to participate in one of two online consensus meetings, timed to optimize participation across global time zones. Each meeting was moderated by three research team members. Meetings were digitally recorded and transcribed. Participants were reminded that the purpose of the meetings was to define a minimum data set for studies of LTS without suggesting new outcomes or considering how an outcome would be measured. They were also reminded that the development of the COS‐LTS would not exclude researchers from collecting extra outcomes in addition to the core set.

For inclusion in the discussion at the consensus meetings, as per COMET guidance [20] outcomes were those rated as 7–9 (critical for inclusion) by ≥ 70% of all participants and 1–3 (not important) by ≤ 15% of all participants. We used the modified nominal group technique [21] to discuss, rank, and combine outcomes with visual facilitation using Google Jamboard. In the second meeting, results of the first consensus meeting were presented to the group toward the end of the session to ensure both unbiased discussion but also final consensus across both groups. As a final step, outcomes proposed for the COS‐LTS were provided to consensus group participants in a Qualtrics survey to state yes/no agreement for inclusion. We selected a deductive criterion [22] of ≥ 50% participants voting yes for COS‐LTS inclusion.

## 3 Results

**FIGURE 1:**
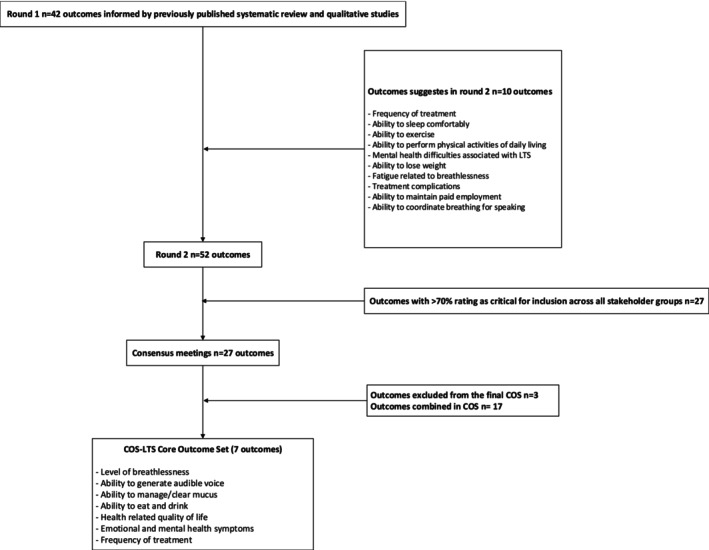
Outcome selection process. COS, Core Outcome Set; LTS, laryngotracheal stenosis.

Figure 1 summarizes the outcome selection process. Our initial list of outcomes for Delphi Round 1 comprised 42 items (Figure 1), (see Table 1). We recruited 1067 participants to the Delphi International expert panel: 974 (91.2%) people living with LTS or family member/friend, 68 (6.0%) clinician researchers, 16 (1.0%) clinicians, and 9 (0.8%) researchers. Participant characteristics are presented in Table 2. Of these 42 outcomes, 14 (33.3%) met consensus criteria by all four stakeholder groups (Table 1). Ten outcomes were added by participants (Figure 1), and therefore, a total of 52 outcomes were voted on twice across the modified Delphi process. Item generation and reduction for the additional outcomes are described in Table S1. In round 2, there were 575 participants, 53.8% of the round 1 participants: 39 (6.8%) clinician researchers, 12 clinicians (2.1%), 518 people living with LTS (90.1%), and 6 researchers (1.0%). From the two rounds of voting, 27 outcomes met the criterion (Table 3) and were taken forward to the consensus meetings. Scores across all outcomes and groups are described in Table S2. Seventeen experts attended the two consensus meetings, including 6 (35.2%) people living with LTS (Table 2).

**TABLE 1:** Delphi round 1 items and scores.

Outcomes	Overall *n* = 1067		Person living with LTS or friend/family (*n* = 974)	Clinical (*n* = 16)	Clinical and research (*n* = 68)	Research (*n* = 9)
	Median (IQR)	% critical	% critical	% critical	% critical	% critical
Breathlessness	9 (7–9)	93	93	87	95	71
Pain or discomfort related to symptoms	7 (5–8)	56	56	60	54	43
Volume of mucus	6 (5–7)	67	69	27	34	43
Viscosity of mucus	6 (5–7)	67	70	20	36	29
Frequency of coughing	6 (5–8)	70	73	27	39	43
Fatigue related to voice use	6 (4–7)	51	52	27	42	43
Fatigue related to swallowing	6 (4–8)	38	37	33	44	29
Negative emotions associated with inability to use voice	6 (5–7.5)	46	45	47	53	43
Positive emotions associated with ability to use voice	6 (4–8)	42	42	40	47	57
Negative emotions associated with the inability to swallow	6 (4–8)	40	39	40	51	43
Positive emotions associated with the ability to swallow	6 (4–8)	35	35	33	47	43
Ability to use voice at work	8 (5–9)	75	76	73	63	63
Ability to communicate care, comfort, and safety needs	7 (6–9)	72	71	93	79	50
Ability to gain attention (e.g., of family member/work colleague)	7 (5–8)	57	56	73	68	56
Ability to participate in and direct a conversation (i.e., two‐way exchange of information)	7 (5–9)	70	70	67	71	63
Ability to use voice to participate in social activities	7 (5–9)	64	65	53	59	63
Ability to raise voice	6 (5–8)	47	48	40	34	63
Ability to sing	5 (3–6)	20	21	0	9	38
Ease/effort of using voice	7 (5–7)	61	64	27	34	63
Ability to generate audible voice	7 (5.5–8.5)	70	71	60	66	63
Voice intelligibility	7 (5–8)	67	67	60	61	75
Ability to communicate at a normal volume	7 (5–9)	61	63	53	32	63
Ability to use a spoken pitch consistent with your identity	6 (4–7)	45	46	33	25	50
Ability to use a consistent voice	6 (4–8)	53	54	33	32	50
Ability to use voice‐activated technology	5 (3–7)	22	22	7	21	63
Ability to eat and drink socially	7 (5–8)	51	50	67	66	50
Ability to eat and drink without clearing mucus	6 (4–8)	59	61	33	36	50
Ability to drink thin fluids	7 (4–8)	52	51	73	55	50
Ability to eat and drink usual food consistencies/textures	7 (4–8)	56	57	73	46	50
Ability to eat and drink without worrying about coughing or choking	7 (5–8)	70	70	80	64	63
Ability to meet nutritional needs without supplements	6 (5–8)	41	40	53	55	88
Health‐related quality of life	8 (6–9)	83	83	73	79	100
Perceived health status	7 (5–8)	63	64	67	50	63
Ability to breathe without stridor	8 (6–9)	88	89	87	79	88
Ability to clear mucus easily	8 (6–9)	87	89	73	61	75
Ability to live without a tracheostomy	8 (7–9)	84	84	100	82	88
Ability to live without tube feeding	9 (7–9)	81	81	87	77	75
Management of symptoms as an outpatient	7 (5–9)	73	74	38	69	75
Management of symptoms as a day case	7 (5–9)	61	62	25	52	88
Patient/carer burden	7 (5–9)	56	55	56	62	88
Mucus plugs	8 (3.25–9)	77	78	73	72	75
Emergency department attendance	8 (5–9)	66	65	73	79	75

Abbreviation: LTS, laryngotracheal stenosis.

**TABLE 2:** Participant characteristics.

Delphi (*n* = 1067)	*n* (%)
Sex (M)	47 (4.4)
Age (years)	
< 30	21 (2.0)
30–50	436 (40.9)
> 50	610 (57.1)
Country of Residence	
The United States of America	657 (61.6)
The United Kingdom	124 (11.6)
Canada	119 (11.3)
Australia or New Zealand	107 (10.0)
Europe	57 (5.3)
Other	3 (0.2)
Involvement with laryngotracheal stenosis (LTS)	
Person Living with LTS or family member/friend	974 (91.2)
Clinician and researcher	68 (6.0)
Clinician	16 (1.0)
Researcher	9 (0.8)
Profession of healthcare/research participants^a^	
Surgeon/Physician	57 (41.0)
Other healthcare professional	24 (17.2)
Nurse	21 (15.1)
Speech Language Therapist	15 (10.8)
Researcher (nonhealthcare professional)	11 (7.9)
PhD (no‐healthcare professional)	6 (4.3)
Physiotherapist	5 (3.6)
Years of experience working with LTS^b^	
< 2 years	5 (5.4)
2 to 5 years	5 (5.4)
5 year	59 (63.4)
Not completed	24 (25.9)

Consensus meetings (*N* = 17)	*n* (%)
Sex (M)	5 (29.4)
Person Living with LTS or family member/friend	6 (35.2)
Clinician	5 (29.4)
Clinician and researcher	6 (35.2)

^a^ Data complete for 139 participants, some of whom coidentified as a clinician or researcher AND a person living with LTS or a family member/friend; percentages represent n/139.

^b^ Data complete for 93 clinicians, researchers, and clinician researchers; percentages represent n/93.

**TABLE 3:** Round 2 Delphi scores meeting COS inclusion criteria.

Outcomes‐Overall participants (*n* = 575)	Median (IQR)	Critical (%)
Outcome 1 Breathlessness	9 (8–9)	94
Outcome 2 Volume of mucus	7 (6–8)	72
Outcome 3 Viscosity of mucus	7 (6–7)	72
Outcome 4 Frequency of coughing	7 (6–8)	75
Outcome 5 Ability to use voice at work	7 (7–8)	79
Outcome 6 Ability to communicate care/comfort/safety needs	8 (7–9)	76
Outcome 7 Ability to participate in and direct a conversation	7 (7–8)	76
Outcome 8 Ability to use voice to participate in social activities	7 (6–8)	71
Outcome 9 Ability to generate audible voice	7 (7–8)	78
Outcome 10 Voice intelligibility	7 (6–8)	74
Outcome 11 Ability to eat and drink without worrying about coughing or choking	7.5 (7–9)	78
Outcome 12 Health‐related quality of life	8 (7–9)	90
Outcome 13 Ability to breathe without stridor	8 (8–9)	92
Outcome 14 Ability to clear mucus easily	8 (7–9)	93
Outcome 15 Ability to live without a tracheostomy	9 (8–9)	91
Outcome 16 Ability to live without tube feeding	9 (8–9)	90
Outcome 17 Management of symptoms as an outpatient	7 (7–8)	80
Outcome 18 Mucus plugs	8 (7–9)	82
Outcome 19^a^ Frequency of treatment	7 (6–8)	79
Outcome 20^a^ Ability to sleep comfortably	8 (7–8)	89
Outcome 21^a^ Ability to exercise	7 (6–8)	80
Outcome 22^a^ Ability to perform physical activities of daily living	8 (7–9)	94
Outcome 23^a^ Mental health difficulties associated with LTS	7 (7–8)	75
Outcome 24^a^ Fatigue related to breathlessness	7 (6–8)	81
Outcome 2^a^ Treatment complications	8 (7–9)	78
Outcome 26^a^ Ability to maintain paid employment	7 (7–8)	76
Outcome 27^a^ Ability to coordinate breathing for speaking	7 (7–8)	87

Abbreviations: COS, core outcome set; LTS, laryngotracheal stenosis.

^a^ 420 participants voted on this outcome (35 clinician researchers,12 clinicians, 371 people living with laryngotracheal stenosiss or their family/friends, 2 researchers).

Outcomes related to breathlessness and stridor were unanimously considered of highest importance for inclusion in the COS, matching the e‐Delphi voting rounds, with consensus in the groups that these could be captured as a single outcome defined as “level of breathlessness.” Similarly, outcomes relating to mucus, voice, and swallowing were collapsed into three single outcomes defined as “ability to generate audible voice,” “ability to manage/clear mucus,” and “ability to eat and drink.” “Health related quality of life” was agreed as an umbrella outcome that accounted for outcomes related to functional status, for example, exercising and sleeping. However, there was a strong preference, particularly from people living with LTS, for “emotional and mental health symptoms” to be a separate outcome due to the emotional distress they described because of their condition. The final reduction of outcomes focused on how to capture management of LTS symptoms with different measurement parameters referenced in the consensus process—how often LTS symptoms need managing, the location where LTS symptoms are managed (e.g., hospital or home), and by whom (e.g., self‐management or clinician). The final descriptor for this outcome was defined as “frequency of treatment,” as there was clear consensus that this was the most important feature of clinical management across stakeholder groups. The list of seven outcomes was voted on by the 17 participants with 82.4% agreement on the final COS. Therefore, the COS‐LTS comprises (1) Level of breathlessness, (2) Ability to generate audible voice, (3) Ability to manage/clear mucus, (4) Ability to eat and drink, (5) Health‐related quality of life, (6) Emotional and mental health symptoms, and (7) Frequency of treatment (Figure 1).

## 4 Discussion

This study establishes a COS comprising seven outcomes for use in intervention studies focused on LTS in adults. We have followed COMET methods for COS development, including item generation via a systematic review and qualitative studies investigating the lived experience of LTS and a multistep consensus phase. These should be used in future LTS studies to allow for direct comparison and standardized reporting of outcomes.

“Level of breathlessness” was the outcome that achieved the highest consensus across and between groups at every stage of the process. There is a similar focus in the evidence base on improving breathing and reducing stridor as the primary outcome of treatment [10, 23]. Consideration of how interventions impact the ability to breathe is consistent with the lived experience of people with LTS who are unable to carry out basic activities of daily living when their symptoms are severe. Inclusion of the level of breathlessness is consistent with COS from other chronic illnesses that impact breathing, such as chronic obstructive pulmonary disorder (COPD) and heart failure [24, 25].

Surprisingly, the need for tracheostomy was not selected for the COS. From free text comments in the e‐Delphi and during the consensus group discussions, this outcome was felt to be important but not as critical to include as breathlessness, partly because, unlike difficulty breathing, a tracheostomy was not relevant for all people living with LTS. Using the online iSGS support group as a recruitment source was likely a factor in the need for tracheostomy not being selected for the final COS. However, not all participants were recruited via this group, and the final consensus discussion was made up of clinicians and researchers as well as people living with iSGS. Consensus discussions were directed toward fundamental outcomes relevant to all people living with LTS, both those with iSGS and other etiologies. The need for tracheostomy was not felt to be necessary for inclusion in every study of LTS and therefore was not selected as a core outcome. This does not prevent tracheostomy from being added as an additional outcome to studies using the COS if it was key to a specific intervention. There was also agreement that the presence or absence of a tracheostomy could be used as a surrogate measure for breathlessness. Dyspnea measures are also used as a surrogate in existing research [9, 26], but this will need to be explored further in the next steps following COS development—when the best tools to measure the outcomes are considered.

The inclusion of “ability to generate audible voice,” “ability to eat and drink,” and “ability to clear mucus” within the COS aligns with previous qualitative research findings [15, 16, 17]. Similarly to these original studies, voice and mucus were more clearly identified as critical, but an outcome related to swallowing was still judged to be necessary for inclusion due to the potential distress and life impact if eating and drinking were disrupted because of an intervention for LTS. Existing intervention studies in LTS are beginning to consider secondary functional outcomes when assessing effectiveness [8, 27, 28] and in the future, the use of the COS‐LTS will support this. The inclusion of functional outcomes allows the COS‐LTS to be applicable to research that includes both surgical and behavioral interventions. This aligns with the wider healthcare evidence base that recognizes breadth in outcomes is necessary for detailed understanding of the effects of an intervention, but also to allow for applicability to clinical care [29, 30].

There was acknowledgment throughout the consensus process that health‐related quality of life (HRQOL) is a challenging and profoundly individual concept to define. However, there was agreement that it offers a useful umbrella term to capture a range of concepts that may relate to LTS outcomes, with existing studies demonstrating the negative impact LTS has on HRQOL [31]. There was a clear consensus, however, that emotional and mental health symptoms should be measured separately from HRQOL because of the emotional distress LTS can cause [32].

“Frequency of treatment” was identified as a core outcome, with other aspects of clinical management of symptoms not meeting consensus, since different stakeholders placed different priorities on these. By comparison, frequency was defined as a non‐negotiable outcome to be included in the COS, particularly in relation to surgical procedures, with all parties agreeing that reducing frequency was an important outcome to demonstrate that an intervention had an impact. In future studies, researchers will be welcome to include additional outcomes they believe to be meaningful and relevant to their intervention, alongside the COS‐LTS, for example, presence of tracheostomy for intervention studies relating to iatrogenic LTS or self‐management of symptoms for studies relating to iSGS.

The purpose of this study was to define the concepts necessary to include in a COS, as per COMET [11] methodology. Outcomes included in this COS have measures described in the LTS evidence base, including clinical trials [33, 34]. These include the Voice Handicap Index‐10, surgical dilation interval, Eating Assessment Tool‐10, and Peak Expiratory Flow. To our knowledge, the outcome “ability to clear mucus” does not have an existing measurement tool. However, the purpose of this study was to gain consensus on the outcomes to be measured and not to recommend or define specific outcome measures at this stage. The next steps for this work relate to determining the appropriate measurement characteristics for each outcome within the COS, including consideration of whether existing tools are fit‐for‐purpose or the need to develop new, psychometrically robust measures.

### 4.1 Strengths and Limitations

Strengths of this study include the adherence to COMET and COS‐STAD reporting guidelines [11, 12] to follow a systematic and structured approach to COS development, as well as a systematic assessment of quality following published guidelines [13]. Our expert Delphi stakeholder panel was international and had excellent patient stakeholder engagement, with people living with LTS or their family/friends representing the largest group within the e‐Delphi process, likely because of engagement with an online support group for people living with iSGS. This means that this COS is representative of the outcomes that matter to patients, given the chronic nature of LTS, as well as the professionals developing intervention studies.

One study limitation is that we did not collect data on the etiology of LTS as part of the Delphi study. Participants had to self‐report their LTS diagnosis and duration but not provide the cause. However, the intention of this study was to develop a COS for all LTS intervention studies irrespective of etiology; therefore, this was not prioritized as part of data collection. Due to the extremely high response rate from people living with LTS or their friends/family, other stakeholder groups were less represented in the responses. Selection bias resulted from the proportion of stakeholders with lived experience, particularly those recruited via the online iSGS support group, who represent individuals who are motivated to engage with their condition but can only offer lived experience of iSGS. There was also a very low response rate from men (4%), consistent with known gender issues in engagement with survey‐based research [35, 36], but also indicative of the engagement of women living with iSGS [23]. However, the numbers of clinicians and researchers who participated corresponded to other published consensus studies [22, 37, 38] suggesting that the disparity was indicative of significant engagement with people living with LTS, rather than a lack of engagement from professionals. The two consensus meetings were more balanced in terms of stakeholder representation, including men, which mitigated the potential impact of weighting toward outcomes that were only relevant to one group or etiology of LTS. This is particularly relevant as our aim was to develop a COS that was representative of the heterogeneity of LTS, with the acknowledgment that additional outcomes can be added by researchers as required according to their population of interest. A final limitation is the drop‐off in response rate between round 1 and round 2 of the surveys; however, our round 2 sample size still exceeds that of many published COS.

## 5 Conclusion

The development of this seven‐item COS for LTS will support standardized outcome reporting in future intervention studies of LTS designed to assess and rehabilitate breathing, voice, swallowing, mucus, and cough in adults with LTS. Further work is required to identify the best validated outcome measurement tools. The use of the COS‐LTS will lead to more homogenized outcomes and the ability to combine trial data and identify effective interventions for both researchers and clinicians.

## Conflicts of Interest

The authors declare no conflicts of interest.

## Supporting information


**Data S1.** Core Outcome Set—STAndards for Development: The COS‐STAD recommendations.

**Data S2.** Quality assessment—Delphi Methodology in healthcare research: How to decide its appropriateness.

**Table S1.** Item generation and reduction from Delphi round 1—all additional outcomes that people suggested in Round 1.

**Table S2.** Round 2 Delphi Scores.
