# Supplementary material for: Development of a Core Outcome Set for Intervention Studies in Adults With Laryngotracheal Stenosis
Source: Laryngoscope. 2025 May 12;135(10):3756–65. doi: 10.1002/lary.32262 (PMC12475546; doi:10.1002/lary.32262)
Supplement: Supplementary file 1 — Data S1. Core Outcome Set—STAndards for Development: The COS‐STAD recommendations. [file LARY-135-3756-s004.docx]

**Delphi methodology in healthcare research: How to decide its appropriateness**

World J Methodol. 2021 Jul 20;11(4):116–129. doi: 10.5662/wjm.v11.i4.116

| **Evaluation Point** | **Score** |
| --- | --- |
| Identification of Problem Area | Yes |
| Selection of Panel Members | Yes (predefined selection criteria) |
| Anonymity of Panelists | Yes (survey), No (panelists*) |
| Controlled Feedback | Yes |
| Iterative Rounds | Yes |
| Consensus Criteria | Yes |
| Analysis of Consensus | Yes |
| Closing Criteria | Yes (panel*) |
| Group Stability | Yes |
| Number of Rounds | 2 |
| Number of Experts | 575 |

*The original article does not relate to the use of Delphi methodology for the development of core outcome sets and therefore does not consider use of a consensus panel as part of the process.
