# Supplementary material for: Development of a Core Outcome Set for Intervention Studies in Adults With Laryngotracheal Stenosis
Source: Laryngoscope. 2025 May 12;135(10):3756–65. doi: 10.1002/lary.32262 (PMC12475546; doi:10.1002/lary.32262)
Supplement: Supplementary file 2 — Data S2. Quality assessment—Delphi Methodology in healthcare research: How to decide its appropriateness. [file LARY-135-3756-s003.docx]

Core Outcome Set—STAndards for Development: The COS-STAD recommendations.

<https://doi.org/10.1371/journal.pmed.1002447.t002>

| **Domain** | **Standard Number** | **Methodology** | **Location in manuscript** |
| --- | --- | --- | --- |
| Scope Specification | 1 | The research or practice setting(s) in which the COS is to be applied | Yes, introduction, page 7, line 55-57 |
|  | 2 | The health condition(s) covered by the COS | Yes, introduction, page 6 48-52 page 7, line 55-57 |
|  | 3 | The population(s) covered by the COS | Yes, introduction, page 6 48-52 & page 7, line 55-57 |
|  | 4 | The intervention(s) covered by the COS | Yes, introduction, page 7, line 55-57 |
| Stakeholders involved | 5 | Those who will use the COS in research | Yes, methods, page 8, line 86-91 |
|  | 6 | Healthcare professionals with exerience of patients with the condition | Yes, methods, page 8, line 86-91 |
|  | 7 | Patients with the condition or their representatives | Yes, methods, page 8, line 86-91 |
| Consensus process | 8 | The initial list of outcomes considered both healthcare professionals’ and patients’ views | Yes, methods, page 7 65-76 |
|  | 9 | A scoring process and consensus definition were described a priori | Yes, methods, page 10, 130-132 |
|  | 10 | Criteria for including/dropping/adding outcomes were describe a priori | Yes, methods, page 9, 112-113 and methods 10, 130-132 |
|  | 11 | Care was taken to avoid ambiguity of language used in the list of outcomes. | Yes, methods, page 7 73-76 |
