# Supplementary material for: Development of a Core Outcome Set for Intervention Studies in Adults With Laryngotracheal Stenosis
Source: Laryngoscope. 2025 May 12;135(10):3756–65. doi: 10.1002/lary.32262 (PMC12475546; doi:10.1002/lary.32262)
Supplement: Supplementary file 3 — Table S1. Item generation and reduction from Delphi round 1—all additional outcomes that people suggested in Round 1. [file LARY-135-3756-s002.docx]

Supplementary Table 1: Item generation and reduction from Delphi round 1 –all additional outcomes that people suggested in Round 1

| **Outcomes Presented** | **COS inclusion** | **Rationale** | **Final outcome wording for Delphi inclusion** |
| --- | --- | --- | --- |
| - Frequency of surgical treatment - Treatment reduces frequency of future surgeries - Number of months without symptoms after intervention - Number of months with symptoms; not requiring surgery - Duration of treatment efficacy (how long before treatment needs to be repeated) - Increased time between dilations - Preventive care of recurrence (following surgery) - Length of time between treatments - Frequency of dilations - time between treatments - The ability to improve over time. For example: going longer between treatments - The rate of scar tissue buildup - time factor - Slowing the formation of the scar tissue - Frequency of treatment in OR - Frequency of injections needed - Able to keep trachea open without dilation - Avoid dilation surgeries | INC-MOD | Captures a unique concept not previously included. | Frequency of treatment (surgery or other) |
| - Ability to exercise - Ability/Limitation to exercise to maintain health - Able to engage with exercise - Ability to maintain fitness / exercise regime - Ability to participate in regular exercise to stay fit/healthy without frustration or difficulty due to stenosis - Ability to exercise e.g. brisk walk / uphill walk - Ability to exercise as desired - Ability to partake in exercise for general and heart health - Ability to exercise safely; climb stairs - Ability to participate in physical exercise without coughing - Number of steps per day - Being able to exercise without shortness of breath - Exercise tolerance regarding leisure activities - My general level of fitness deteriorated due to stridor and respiratory distress. Certain activities were beyond me but other activities were manageable. I think assessing fitness level is a good outcome to consider. Exercise tolerance regarding athletic activities - Ability to participate fully in sports activities - Ability to participate in activities - Ability to participate in regular physical activity - Ability to run/sport activity - Ability to perform activities of daily living and moderate exercise - Ability to engage in activities such as walking; yoga; or other low-impact exercise without fear - Ability to exercise frequently - Being able to walk - Being able to climb stairs - Being able to be physically active- work out without losing breath or needing to pause cardio - Ability to maintain a moderate exercise regime - Mucous production during exercise - Mucous production interfering with exercise - Ability to engage in physical activity / exercise - Ability to exercise comfortably - Ability to walk briskly or climb hills without shortness of breath - Oxygen saturation while walking - The ability to breathe fully when active or exercising. - Ability to easily undertake exercise | INC  INC-MOD | Captures a unique concept not previously included. | Ability to exercise |
| - Ability/Limitation to travel by public transport (e.g. aeroplane) | REDUN | Not an outcome relevant to this study |  |
| - Ability to explain my condition to others | REDUN | Not an outcome |  |
| - Limit antibiotic usage/ appropriate use of antibiotics | REDUN | Overlaps with frequency of treatment and management of symptoms |  |
| - Ability to be seen within a day of change of symptoms, i.e. possible infection | REDUN | Overlaps with management of treatment |  |
| - Possibility of further treatment without general anaesthetic | REDUN | Captured by management as an outpatient |  |
| - Speed of recovery after treatment | REDUN | Captured by perceived health status |  |
| - Ability to walk or any physical activity so that you do not choke or cough from mucus - Able to perform physical activities of daily living - Able to perform physical activities for job - Ability to undertake routine household tasks - Ability to undertake other physical work - Not to become short of breath with minimal activity - being able to maintain ADLS without becoming short of breath - Ability to participate in activities without feeling dizzy or lightheaded - Exercise tolerance regarding activities of daily living - Monitoring activities of daily living (ADL) - Ability to have sex with partner due to airway - Ability to perform activities of daily living and moderate exercise - Ability to climb a flight of steps and not stop and catch breath - Being able to perform basic life needs such as cleaning/washing - Being able to carry anything - Ability to complete activities of daily living - Ability to complete normal activities of daily living without shortness of breath - Ability to climb stairs without shortness of breath - Ability to do normal things (e.g. cleaning; gardening) without shortness of breath - Ability to perform ADLs without restriction from dyspnoea - Ability to move without becoming short of breath - Ability to walk up stairs without experiencing stridor - Ability to do tasks in daily life that you find important or fulfilling - Ability to complete daily tasks | INC-MOD | Mucus aspect captured by ability to clear mucus easily; new aspect of outcome captured with alternative wording | Ability to perform physical activities of daily living |
| - Sleeping with breathing issues - Ability to have uninterrupted sleep due to coughing/choking - Ability to breather easily when lying down - Monitoring sleep patterns - Ability to sleep comfortably - Sleep improvement - tiredness/ quality of life is most affected by disturbed sleep - Sleep disturbance due to presence of post nasal drip/mucous clearing | INC-MOD | Captures a unique concept not previously included. | Ability to sleep comfortably |
| - Quick and ACCURATE diagnosis - Ability to have diagnosis prior to extreme breathlessness - Easy access to doctors educated about this disease - Confidence in surgeon; healthcare provider knowledge and expertise - Medical training and awareness of diagnosis - Health care professionals having more awareness of this condition - Ability to get an accurate diagnosis | REDUN | Not an outcome, linked to question of diagnosis and breathlessness already captured |  |
| - Avoid social discomfort of audible wheeze - Impact on mental health and well being when not breathing well - Mental health and well being supports for long term health condition - Loss of confidence and personality due to voice loss - Increased anxiety/depression - Fear of being unable to breathe - Level of anxiety regarding when symptoms will reoccur - PTSD regarding previous diagnosis and treatment - Psychological well being - Worry about long term effects - Medical trauma; depression/anxiety related to condition - Mental health support (with living with a life long debilitating disease) - Improved attention capacity - Positive mental health regarding diagnosis - Mental health with long no term air way issues. - Mental Health related to living with chronic disease (not specific to voice; swallow; etc. - Ability to participate in family and social life -level of self-consciousness with symptoms occurring in public -ability to fully engage in recreational activities and hobbies | INC-MOD |  | Mental health difficulties associated with LTS |
| - Much higher recognition in UK for action in management of mucus issues | REDUN | Not an outcome |  |
| - Losing weight is a big factor in helping breathing. | INC-MOD | Not previously captured in existing outcomes | Ability to lose weight |
| - Keep walking each day and try and live a normal life. | REDUN | Not an outcome |  |
| - Ability to be managed at home without care | REDUN | Already captured by several existing outcomes e.g. patient and carer burden, health status |  |
| - Need for tracheal suction to clear secretions | REDUN | Not an outcome |  |
| - Shortness of breath | REDUN | Already included in breathlessness |  |
| - Limit number of days voice is impacted after dilation or other procedure | REDUN | Not an outcome |  |
| - Fatigue in general; not just from voice use or swallowing but just trying to breathe - Fatigue from day to day activities (breathing; walking; stairs) - Fatigue - even when airway open pre-resection fatigue was ever present. It is no more post-resection - Improved tiredness levels | INC-MOD | Not previously captured in existing outcomes | Fatigue related to breathlessness |
| - Confidence in provider/doctor for management and /or surgical needs - Access to a provider/doctor for magement and/or surgical needs | REDUN | Not an outcome |  |
| - Complications of procedures - Complications after dilation - Complications from chronic coughing - Complications from treatment options - Complications tearing/emphysema (rare but do occur) - Extra damage caused to airway during treatment e.g. to vocal cords during laser/dilation treatment - Scar in the neck - Chipped teeth | INC-MOD | Not captured in existing outcomes | Treatment complications |
| - Vocal Cord Dysfunction in relation to ISS patients | REDUN | Not an outcome |  |
| - Daily living with Nebulizer machine to clear mucous. | REDUN | Not an outcome |  |
| - Side effects of treatment that negatively impact quality of life | REDUN | Captured by several existing outcomes including Health related quality of life |  |
| - Patient perception of value of treatment versus negative side effects | REDUN | Captured by several existing outcomes including Health related quality of life and patient burden |  |
| - Recovery time after dilation | REDUN | Not an outcome |  |
| - Ability to speak in a crowded setting | REDUN | Already captured by other outcomes related to voice e.g. ability to raise voice. |  |
| - Communicate with strangers rather than avoid them due to excessive clearing of throat or having to explain the stridor. | REDUN | Not an outcome, some concepts captured elsewhere. |  |
| - Ability to play the flute semi-professionally | REDUN | Not an outcome |  |
| - Able to manage condition without side effects from medication | REDUN | Not an outcome |  |
| - Peak flow readings - Change in Peak Flow - Change in peak flow metre readings - Peak flow of breathing - Increase in peak flow - Peak inspiratory flow rate as measured by pulmonary function testing | REDUN | Outcome measure, not outcome for COS |  |
| - Ability to maintain employment due to decline in health or voice quality - Ability to change or regain employment whilst having laryngotracheal stenosis symptoms (loss of voice) - Minimize the amount of time taken off of work to monitor condition - Inability to work/ support getting disability status or workplace accommodations | INC-MOD | Not captured in existing outcomes | Ability to maintain paid employment |
| - Excessive coughing causing bladder incontinence | REDUN | Not an outcome |  |
| - Reduction of cognitive faculties | REDUN | Not an outcome |  |
| - Standardization | REDUN | Not an outcome |  |
| - Increase in breathlessness while talking | REDUN | Already captured in existing outcomes e.g. breathlessness. |  |
| - Flow cytometry reading | REDUN | Outcome measure, not outcome for COS |  |
| - Stenosis due to sarcoidosis | REDUN | Not an outcome |  |
| - Change in radiologic Appearance in Subglottic/Tracheal Scar | REDUN | Outcome measure |  |
| - Change in Dyspnea Patient Reported Outcomes (CCQ or DI) | REDUN | Outcome measure |  |
| - Cepstral Peak Prominence | REDUN | Outcome measure |  |
| - Mean Phonation Time - Maximal duration of phonation | REDUN | Outcome measure |  |
| - Financial support for suffers | REDUN | Not an outcome |  |
| - Being able to breathe quietly/normally | REDUN | Already captured in existing outcomes e.g. breathlessness, breathe without stridor |  |
| - Being able to talk without becoming short of breath. | REDUN | Already captured |  |
| - Level of social comfort regarding coughing and loud breathing etc when in public or at work | REDUN | Already captured elsewhere |  |
| - Ability to access medical care in a timely fashion | REDUN | Not an outcome |  |
| - It is very uncomfortable to always to have the need to clean our throats and gasp for air | REDUN | Not an outcome |  |
| - Patients’ treatment preference - right to choose a resection as an early option | REDUN | Not an outcome |  |
| - Ease of breathing- again post resection is completely different to post balloon dilatation | REDUN | Already captured in existing outcomes e.g. breathlessness |  |
| - Muscle spasms in extremities | REDUN | Not an outcome |  |
| - Able to breathe deeply and fully without coughing. | REDUN | Already captured in existing outcomes e.g. breathlessness |  |
| - To stop scarring from returning | REDUN | Not an outcome |  |
| - In office steroid injections and if they help with regrowth | REDUN | Not an outcome |  |
| - Ability to access health providers that are familiar with the condition and can effectively treat it | REDUN | Not an outcome |  |
| - Impact of reduced airflow on other organs such as heart | REDUN | Not an outcome |  |
| - Ability to deal with financial stress of this disease. | REDUN | Not an outcome although linked to mental health and ability to work |  |
| - Acquiring infections in throat and sinuses | REDUN | Not an outcome |  |
| - Management of GERD and LPR | REDUN | Not an outcome |  |
| - Ability to speak a full sentence (set number of syllables) in one breath | REDUN | Already captured in existing outcomes e.g. breathlessness |  |
| - Ability to walk at a moderate pace and converse simultaneously | INC-MOD | Ability to coordinate breathing for walking and talking |  |
| - Changes to reaction to cold weather on airway | REDUN | Not an outcome |  |
| - Ability to have first responders be aware of the stenosis in an emergency (when patient may not be able to communicate) | REDUN | Not an outcome |  |
| - Ability to contact doctor and have dilation in a timely manner | REDUN | Not an outcome |  |
| - Emergency rooms and ENT/Endocrinologist should have to be trained on this disease immediately | REDUN | Not an outcome |  |
| - Having instant access to health care professionals | REDUN | Not an outcome |  |
| - Alleviating mucus | REDUN | Already captured in existing outcomes e.g. volume of mucus |  |
| - Memory issues from anesthesia | REDUN | Not an outcome |  |
| - Covid vaccine - Covid infection | REDUN | Not an outcome |  |
| - Ability to receive specialist care with aim to promote good voice quality | REDUN | Not an outcome |  |
| - Ability to find a knowledgeable health care provider | REDUN | Not an outcome |  |
| - Increased pt confidence and less delay in reporting possible complications and concerns to dr and staff re: possible complications and serious questions; | REDUN | Not an outcome |  |
| - The ability to live without severe heartburn | REDUN | Not an outcome |  |
| - Level of patients’ markers for chronic inflammation? | REDUN | Not an outcome |  |
| - Finding a genetic link or ability to test (I have 4 family members and 3 generations with disease) | REDUN | Not an outcome |  |
| - The role of hormone fluctuations with symptoms and daily life with disease (stridor/mucous worsening at different time of cycle) | REDUN | Not an outcome |  |
| - If other parts of the body are affected by fibrotic lesions or growths or stenosis in other parts of the body | REDUN | Not an outcome |  |
| - How many people also have endometriosis in conjunction with stenosis symptoms | REDUN | Not an outcome |  |
| - Climate and environmental factors that can potentially contribute to populations with many patients living with stenosis ( eg: Saskatchewan and Alberta) | REDUN | Not an outcome |  |
| - Dyspnea Index | REDUN | Outcome measure |  |
| - Urinary Incontinence as a result of excessive coughing | REDUN | Not an outcome |  |
| - Maximal voice volume | REDUN | Outcome measure |  |
| - Access to correct treatment | REDUN | Not an outcome |  |
| - Ability to manage symptoms on a daily basis | REDUN | Captured elsewhere e.g. health related quality of life. |  |
| - Ability to know when you need treatment | REDUN | Not an outcome |  |

Each • indicates one outcome suggestion; INC-MOD = included but wording modified; REDUN = redundant/overlapping outcome; INC = include as is.
