# Supplementary material for: Development of a Core Outcome Set for Intervention Studies in Adults With Laryngotracheal Stenosis
Source: Laryngoscope. 2025 May 12;135(10):3756–65. doi: 10.1002/lary.32262 (PMC12475546; doi:10.1002/lary.32262)
Supplement: Supplementary file 4 — Table S2. Round 2 Delphi Scores. [file LARY-135-3756-s001.docx]

**Supplementary Table 2 Round 2 Delphi Scores**

| **Outcomes** | **Overall**  **(*N* = 575)** | | **Person living with LTS or friend/family**  **(n = 518)** | **Clinical**  **(n = 12)** | **Clinical and research (n=39)** | **Research**  **(n=6)** |
| --- | --- | --- | --- | --- | --- | --- |
|  | **Median (IQR)** | **% critical** | **% critical** | **% critical** | **% critical** | **% critical** |
| Breathlessness ^b^ | 9 (7-9) | 94 | 94 | 92 | 92 | 83 |
| Pain or discomfort related to symptoms | 6 (7-8) | 61 | 61 | 75 | 59 | 83 |
| Volume of mucus^b^ | 7 (6-8) | 72 | 75 | 25 | 44 | 50 |
| Viscosity of mucus^b^ | 7 (6-7) | 72 | 76 | 8 | 44 | 50 |
| Frequency of coughing^b^ | 7 (6-8) | 75 | 78 | 33 | 49 | 83 |
| Fatigue related to voice use | 6 (5-7) | 56 | 58 | 17 | 44 | 67 |
| Fatigue related to swallowing | 6 (5-7) | 37 | 36 | 33 | 38 | 67 |
| Negative emotions associated with inability to use voice | 7 (6-7) | 49 | 49 | 33 | 54 | 83 |
| Positive emotions associated with ability to use voice | 6 (5-7) | 42 | 42 | 25 | 44 | 67 |
| Negative emotions associated with inability to swallow | 6 (5-8) | 41 | 41 | 25 | 46 | 50 |
| Positive emotions associated with ability to swallow | 6 (5-7) | 35 | 35 | 17 | 46 | 17 |
| Ability to use voice at work^b^ | 7 (7-8) | 79 | 79 | 83 | 75 | 50 |
| Ability to communicate care, comfort and safety needs^b^ | 8 (7-9) | 76 | 74 | 92 | 88 | 67 |
| Ability to gain attention (e.g. of family member/work colleague) | 7 (6-8) | 62 | 61 | 75 | 79 | 100 |
| Ability to participate in and direct a conversation^b^ | 7 (7-8) | 76 | 75 | 67 | 88 | 67 |
| Ability to use voice to participate in social activities^b^ | 7 (6-8) | 71 | 71 | 75 | 75 | 83 |
| Ability to raise voice | 6 (5-7) | 46 | 47 | 33 | 30 | 83 |
| Ability to sing | 4 (3-5) | 18 | 19 | 0 | 3 | 33 |
| Ease/effort of using voice | 6 (5-7) | 67 | 70 | 33 | 38 | 83 |
| Ability to generate audible voice^b^ | 7 (7-8) | 78 | 78 | 83 | 78 | 83 |
| Voice intelligibility^b^ | 7 (6-8) | 74 | 74 | 75 | 70 | 83 |
| Ability to communicate at a normal volume | 6 (6-8) | 67 | 69 | 50 | 44 | 67 |
| Ability to use a spoken pitch consistent with your identity | 6 (5-7) | 43 | 45 | 17 | 21 | 67 |
| Ability to use a consistent voice | 6 (5-7) | 52 | 55 | 25 | 28 | 83 |
| Ability to use voice activated technology | 5.5 (4-6.3) | 18 | 18 | 8 | 15 | 50 |
| Ability to eat and drink socially | 7 (6-7) | 57 | 57 | 67 | 64 | 50 |
| Ability to eat and drink without clearing mucus | 6 (6-8) | 67 | 70 | 42 | 31 | 50 |
| Ability to drink thin fluids | 7 (6-8) | 59 | 57 | 75 | 72 | 50 |
| Ability to eat and drink usual food consistencies/textures | 7 (6-8) | 62 | 63 | 75 | 54 | 33 |
| Ability to eat and drink without worrying about coughing or choking^b^ | 7.5 (7-9) | 78 | 79 | 83 | 74 | 67 |
| Ability to meet nutritional needs without supplements | 6.5 (6-8) | 46 | 45 | 50 | 54 | 83 |
| Health related quality of life^b^ | 8 (7-9) | 90 | 90 | 75 | 92 | 100 |
| Perceived health status | 7 (6-8) | 68 | 68 | 67 | 56 | 83 |
| Ability to breathe without stridor^b^ | 8 (8-9) | 92 | 92 | 100 | 92 | 83 |
| Ability to clear mucus easily^b^ | 8 (7-9) | 93 | 94 | 83 | 79 | 83 |
| Ability to live without a tracheostomy^b^ | 9 (8-9) | 91 | 90 | 100 | 97 | 100 |
| Ability to live without tube feeding^b^ | 9 (8-9) | 90 | 89 | 100 | 95 | 83 |
| Management of symptoms as an outpatient^b^ | 7 (7-8) | 80 | 82 | 17 | 80 | 67 |
| Management of symptoms as a day case | 7 (6-8) | 69 | 70 | 25 | 63 | 83 |
| Patient/carer burden | 7 (6-8) | 60 | 60 | 75 | 63 | 67 |
| Mucus plugs^b^ | 8 (7-9) | 82 | 82 | 83 | 90 | 50 |
| Emergency department attendance | 8 (7-9) | 69 | 67 | 67 | 85 | 67 |
| Frequency of treatment^ab^ | 7 (6-8) | 79 | 82 | 46 | 63 | 50 |
| Ability to sleep comfortably^ab^ | 8 (7-8) | 89 | 89 | 91 | 86 | 50 |
| Ability to exercise^ab^ | 7 (6-8) | 80 | 82 | 64 | 64 | 100 |
| Ability to perform physical activities of daily living^ab^ | 8 (7-9) | 94 | 94 | 100 | 92 | 50 |
| Mental health difficulties associated with LTS^ab^ | 7 (7-8) | 75 | 76 | 82 | 69 | 50 |
| Ability to lose weight^a^ | 6 (5-7) | 52 | 55 | 9 | 39 | 50 |
| Fatigue related to breathlessness^ab^ | 7 (6-8) | 81 | 81 | 42 | 85 | 83 |
| Treatment complications^ab^ | 8 (7-9) | 78 | 78 | 83 | 83 | 33 |
| Ability to maintain paid employment^ab^ | 7 (7-8) | 76 | 75 | 73 | 83 | 100 |
| Ability to coordinate breathing for speaking^a^ ^b^ | 7 (7-8) | 87 | 88 | 82 | 71 | 50 |
|  |  |  |  |  |  |  |

^a^ 420 participants (371 people living with LTS or their family/friends, 12 clinicians, 35 clinician researchers, 2 researchers) voted on this outcome

^b^ Met criteria for consensus meeting
